# Regulation of Human Breast Cancer by the Long Non-Coding RNA *H19*

**DOI:** 10.3390/ijms18112319

**Published:** 2017-11-03

**Authors:** Jordan Collette, Xuefen Le Bourhis, Eric Adriaenssens

**Affiliations:** 1 INSERM U908, 59655 Villeneuve d’Ascq, France; jorkan62@gmail.com (J.C.); xuefen.lebourhis@univ-lille1.fr (X.L.B.); 2 University of Lille, 59655 Villeneuve d’Ascq, France

**Keywords:** lncRNA, *H19*, breast cancer, non-coding RNA

## Abstract

Breast cancer is one of the most common causes of cancer related deaths in women. Despite the progress in early detection and use of new therapeutic targets associated with development of novel therapeutic options, breast cancer remains a major problem in public health. Indeed, even if the survival rate has improved for breast cancer patients, the number of recurrences within five years and the five-year relative survival rate in patients with metastasis remain dramatic. Thus, the discovery of new molecular actors involved in breast progression is essential to improve the management of this disease. Numerous data indicate that long non-coding RNA are implicated in breast cancer development. The oncofetal lncRNA *H19* was the first RNA identified as a riboregulator. Studying of this lncRNA revealed its implication in both normal development and diseases. In this review, we summarize the different mechanisms of action of *H19* in human breast cancer.

## 1. Introduction

Breast cancer is the most common tumor in women and caused 508,000 deaths worldwide in 2011 [1]. Recent advances in molecular classification of this pathology allowed personalized-treatment of patients and better outcomes, like the use of Herceptin in patients with overexpression of human epidermal growth factor receptor 2 (Her-2) [2]. However, some classes of breast cancer such as triple-negative, which is characterized by neither expression of progesterone receptor (PR), estrogen receptor, nor Her-2, remains a poor prognostic for patients. The discovery of new molecular actors involved in the regulation of breast cancer development is essential to improve the management of this disease. During the last decades, plenty of non-coding RNAs have been involved in breast cancer development [3]. The study of non-coding RNAs could lead to the development of new therapeutic strategies and better outcomes for patients with triple negative breast cancer, and more generally to patients with cancer.

The Encyclopedia of DNA Elements (ENCODE) consortium revealed that up to 80% of the human genome is transcribed into functional RNAs, but only 2% of the genome codes for proteins [4,5,6]. RNAs without coding potential are referred to as non-coding RNAs (ncRNAs). Based on theirs lengths, they can be classified into two classes: small ncRNAs (<200 nt) and long ncRNAs (>200 nt). Small ncRNAs include microRNAs (miRs), small interfering RNAs (siRNAs), PIWI-interacting RNAs (piRNAs) or small nucleolar RNA (snoRNAs). miRs, siRNAs, and piRNAs were shown to mainly act as negative regulators of gene expression, whereas snoRNA serves as a guide to induce chemical modification of other RNAs [7]. Recently, Hon et al. identified 19,175 potentially functional lncRNAs in the human genome [8]. The majority of lncRNAs shared similarities with mRNA: they are transcribed by RNA polymerase II; 5′ capped; 3′ polyadenylated; and could be subjected to splicing [9,10]. The lncRNAs are also transcriptionally regulated by transcription factor and epigenetic modifications and their expression is cell-type/tissue specific [8].

Implication of lncRNA is well documented in different pathologies, including neurological diseases [11,12], diabetes [13], and cancers [14]. lncRNAs exert their functions through diverse molecular mechanisms such as acting as scaffolds for RNP complex, decoys for transcriptional factors or microRNAs, RNA interference, targeting of transcriptional factor or chromatin modifier protein to specific genomic loci, transcriptional regulation in *cis* or *trans* [15]. In this review, we will focus on the implication of *H19*, the first identified lncRNA, in human breast cancer.

## 2. *H19* Gene Locus

**Figure 1:**
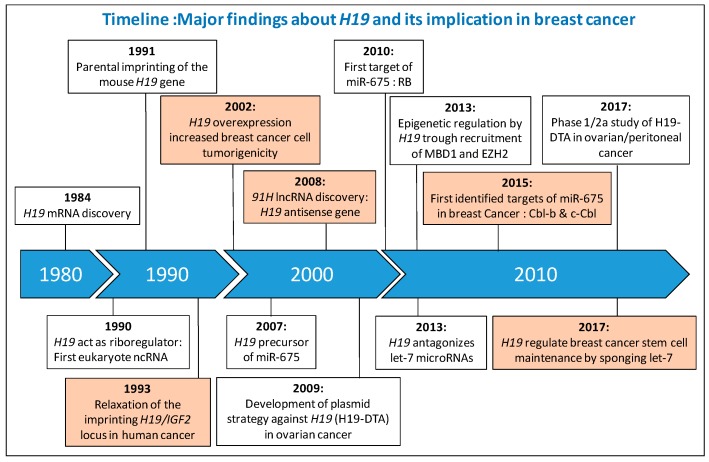
Major finding about *H19* and its implication in breast cancer. Information concerning the implication of *H19* in breast cancer are colored orange. IGF2, Insulin-like Growth Factor 2; lncRNA, Long Non-Coding RNA; RB, Retinoblastoma; MBD1, Methyl-CpG Binding Domain, EZH2, Enhancer of Zeste Homolog 2; Cbl, Casitas B-lineage Lymphoma; H19-DTA, Plasmid encoding the A chain of diphtheria toxin (DT-A) driven by the regulatory sequences of human *H19*.

The *H19*/*IGF2* locus located at position 11p15.5 is subject to genomic imprinting and is encoded for several transcripts. One of these transcripts, *H19*, was proposed to act as a riboregulator by Brannan et al. in 1990 and was the first identified lncRNA [16]. Major findings about *H19* and its implication in breast cancer are summarized below in a timeline manner (Figure 1).

The *H19* gene is transcribed by the RNA polymerase II to give raise a polyadenylated, capped and spliced 2.3 kb RNA. *H19* is paternally imprinted and maternally expressed [17]. It is expressed during embryonic development and repressed after birth expect in a few tissues like mammary gland and uterus [18]. Aberrant expression of *H19* due to imprinting modification is responsible for developmental diseases. The loss of *H19* expression and a biallelic expression of IGF2 are responsible for the Beckwith–Wiedmann syndrome characterized by fetal and postnatal overgrowth and predisposition to pediatric cancers such as Wilm’s tumors [19]. Biallelic *H19* expression and loss of IGF2 expression can lead to Silver–Russel syndrome characterized by intrauterine and postnatal growth retardation combined with others symptoms [19]. In cancer, *H19* is frequently overexpressed and it is associated to many aspect of cancer development [20,21].

In 2007, Cai & Kullen demonstrated that *H19* is a precursor of miR-675 [22]. The two strands of miR-675, miR-675-5p, and miR-675-3p, have been involved in disease development and notably in cancer development (Section 3.1). In the locus *H19*/*IGF2*, we have identified the presence of a new lncRNA antisense to *H19* gene, named *91H* [23]. This lncRNA is also associated with cancer development in solid-tumors such as breast cancer or osteosarcoma [23,24]. The implication of *H19* and *91H* in cancer is well described and is mediated by different mechanisms characteristics of that observed for other non-coding RNAs. In this review, we will focus on the role of *H19* in human breast cancer.

## 3. *H19* Gene Locus in Human Breast Cancer

The implication of *H19* in tumorigenesis has been reported and *H19* is overexpressed in many solid tumors such as prostate, bladder or breast cancers [25,26,27]. We showed that *H19* is overexpressed in 73% of breast cancer tissues when compared to healthy tissues [28]. Several studies showed that *H19* is controlled by steroid hormones in normal and cancerous mammary gland, uterus and prostate [29,30,31]. In breast cancer, the expression of *H19* is higher in Estrogen receptor (ERα) positive cells, but in the ERα negative MDA-MB-231 cell line, ectopic overexpression of *H19* is associated with increased proliferation [27,30]. Collectively, these data indicate that *H19* favor breast cancer development probably thought different mechanisms discussed below.

### 3.1. H19: Precursor of miR-675-5p and miR-675-3p

The *H19*-derived miR-675 gives rise to two functional microRNA, miR-675-5p, and miR-675-3p with different validated targets. The implication of miR-675 in cancer was firstly shown by Tsang WP et al. in colorectal cancer (CRC). By targeting the tumor suppressor retinoblastoma (RB) protein, miR-675-5p regulates the CRC development [32]. In human breast cancer, we have identified two ubiquitin ligase E3, c-Cbl, and Cbl-b, as direct targets of miR-675-5p [33]. The overexpression of miR-675-5p in breast cancer cells lines induced the downregulation of c-Cbl and Cbl-b proteins and increased the stability and the activation of Epidermal growth factor receptor (EGFR) and c-Met. Steady activation of Akt and Erk pathway enhanced the proliferation of human breast cancer and their metastasis abilities in xenograft experiments.

Zhai et al. investigated the expression of miR-675-5p in formalin-fixed paraffin-embedded (FFPE) tissues of 100 breast cancer patients [34]. The authors showed that miR-675 is significantly up-regulated in breast cancer patients compared with controls, but this up-regulation is not correlated with clinical and pathological status including ER and PR expression, age, and lymph node stage. The frequency of miR-675 overexpression was higher in the patients with low histological grade (I and II). Cordero et al. analyzed DNA methylation levels of 517 microRNA encoding genes in prediagnostic peripheral white blood cells of subjects who have developed colorectal cancer or breast cancer (BC) and subjects who remained clinically healthy [35]. They found that eight microRNAs, including miR-675-5p, were differentially methylated in subjects who went on to develop breast cancer. In those subjects, miR-675-5p was significantly hypomethylated suggesting that miR-675-5p could be used as biomarker for breast cancer.

All the known targets of miR-675-5p and miR-675-3p implicated or not in neoplasia are resumed in Table 1.

Some of these targets could explain the oncogenic role of *H19* in breast cancer. For example, miR-675 stimulates migration and invasion by targeting TGF-β1 in prostate cancer cells, Cadherin13 in glioma cells, or RUNX1 in gastric cancer cells [42,44,47]. We found that *H19* and miR-675 expression enhances breast cancer cell migration [33,58]. This could be mediated by targeting the above cited molecules, even if miRNAs targets remain tissue specific. By example, miR-675 was shown to downregulate the expression of RB in human colorectal cancer to promote tumor development [32]. RB was also demonstrated as a target of miR-675-5p in hepatocellular carcinomas [48]. However, we showed that miR-675-5p doesn’t interact with RB mRNA in human breast cancer cell lines [33].

### 3.2. Competing Endogenenous RNAs (ceRNAs): Sequestration of miRs by H19

Tay et al. reported that lncRNA can be in competition with mRNA for common microRNAs and termed such lncRNA transcript as competing endogenous RNAs (ceRNAs) [59]. *H19*, as numerous lncRNAs, could act by this mechanism. Recently, Peng et al. demonstrated the implication of *H19* in maintenance of breast cancer stem cells through the sequestration of let-7 [60]. The lower availability of let-7 increases the expression of its target, the core pluripotency factor LIN28; LIN28 in turn blocks mature let-7 production and enhances the expression of *H19* in breast cancer stem cells. In human breast cancer cells lines, *H19* upregulates the DNA methyltransferase DNMT1 by sponging miR-152, leading to enhancement of cell proliferation and invasion of the cells [61]. The authors also revealed a correlation between the overexpression of *H19* and DNMT1 and the downregulation of miR-152 in human breast tumor tissues. In 2017, Zhou et al. demonstrated that *H19* regulates Epithelial-mesenchymal transition (EMT) and Mesenchymal-epithelial transition (MET) by differentially acting as a sponge for the microRNA miR-200b/b and let-7b using a mouse model of spontaneous metastatic breast cancer [62]. Other microRNAs sequestrated by *H19* are indicated in Table 2.

The impact of *H19* on metastasis abilities of the human breast cancer cell could be due to the sponging of these microRNAs. By example, *H19* regulates EMT in bladder cancer by sponging miR-29b-3p [64]. In thyroid cancer, *H19* promotes proliferation, migration, and invasion of cancer cells through the sponging of miR-17-5p [73]. *H19* also regulates tumorigenic abilities of glioblastoma cells by sponging miR-181-d, in acute myleocytic leukemia by sponging has-miR-19a/b and in osteosarcoma by serving as competing endogenous RNA for the miR-200s family [63,67,72].

**Figure 2:**
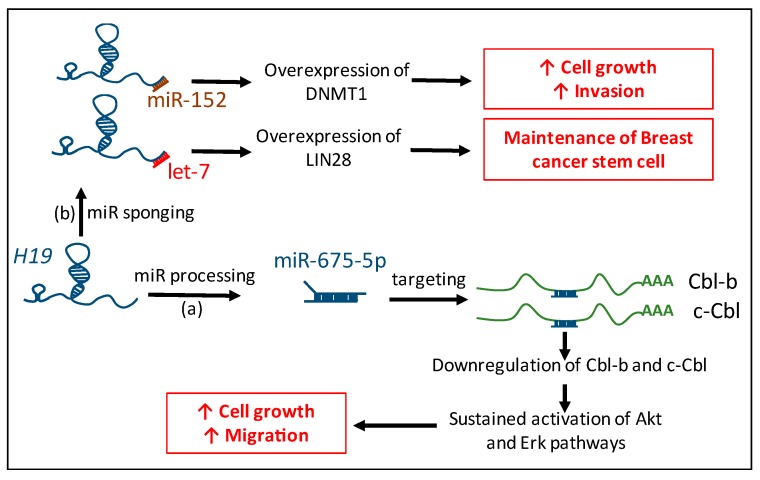
Connection between *H19* and microRNAs. *H19* is the precursor of miR-675-5p which targets Cbl-b and c-Cbl mRNA in breast cancer. Downregulation of Cbl-b and c-Cbl protein expression induces sustained activation of Akt and Erk pathways that lead to increased cell growth and migration potential (a). *H19* physically interacts with miR-152 and let-7 and impairs their bioavailability to induce the overexpression of their targets, DNMT1 and LIN28, and participle in tumorigenic properties and maintenance of stemness in breast cancer cells (b). Red arrows indicate an increased phenotype.

In conclusion, the lncRNA *H19* interacts with miRs pathways not only by being the precursor of miR-675, but also by physically interacting with other miRs to regulate the expression of their targets (Figure 2).

### 3.3. Epigenetics Modification Induced by H19

Long non-coding RNA could interact with chromatin modifier protein and contribute to epigenetic regulation of gene expression. *H19* was shown to interact with the histone methyl transferase Enhancer of zeste homolog 2 (EZH2) and epigenetically silenced *E*-cadherin in bladder cancer and DIRAS3 in diabetic cardiomyopathy [74,75]. Si et al. identified *H19* as a factor associated with paclitaxel resistance in ERα-positive breast cancer cells. *H19* decreases cell apoptosis induced by paclitaxel treatment by inhibiting the transcription of pro-apoptotic genes *BIK* and *NOXA*. The recruitment of EZH2 by *H19* and its targeting onto the promoter of BIK induce its downregulation. As described in Section 3.2, *H19* impairs availability of miR-152 and increases the expression of the epigenetic regulator DNMT1 and so increases proliferation of breast cancer cells lines [61]. However, the epigenetic modification induced by DNMT1 remains unknown. In the embryo, *H19* has been shown to physically interact with MBD1 and induce its recruitment at several imprinted genes including I*GF2*, *PEG1*, and *SLC38A4* [76]. In 2015, Zhou et al. demonstrated that *H19* binds to and inhibits *S*-adenosylhomocysteine hydrolase (SAHH) which carries the synthesis of *S*-adenosylmethionine (SAM), the only source of methyl for methyltransferases and other processes that are methyl-dependent [77]. Modulation of *H19* can modify genome wide DNA methylation and knockdown of *H19* increased DNMT3B-mediated methylation of the lncRNA-encoding gene *Nctc1* within the *Igf2-H19-Nctc1* locus.

Taken together, these data revealed that *H19* is an important regulator of epigenetic status of target genes. The epigenetic regulation driven by *H19* could be done by physical interaction with chromatin modifier protein or indirectly by regulating their expression.

### 3.4. 91H: H19 Antisense Transcript

In 2008, we identified a new non-coding transcript within the *IGF2*/*H19* locus which is an antisense gene to *H19* named *91H* [23]. This transcript of 120 kb in humans is an lncRNA expressed in human and mice from the maternal allele. We also showed that *91H* is overexpressed in breast tumors. Further studies demonstrate the implication of *91H* in osteosarcoma [24] and colorectal cancer [78]. Recently, we showed that in breast cancer cells that *91H* exerts oncogenic properties by promoting cell growth, migration, and invasion [79]. In *91H*-knockdown cell lines the expression of *H19* and IGF2 is reduced through epigenetic modifications on *H19*/*IGF2* locus. These data suggest that *91H* plays an essential role in breast cancer development, but it is possible that *91H* regulation of tumorigenicity requires other factors than *H19* or IGF2. Differentiate the implication of *91H* in biological process independently of *H19* is not an easy task, but well thought experiments are needed to address the issue.

### 3.5. Regulation of Cell Cycle

**Figure 3:**
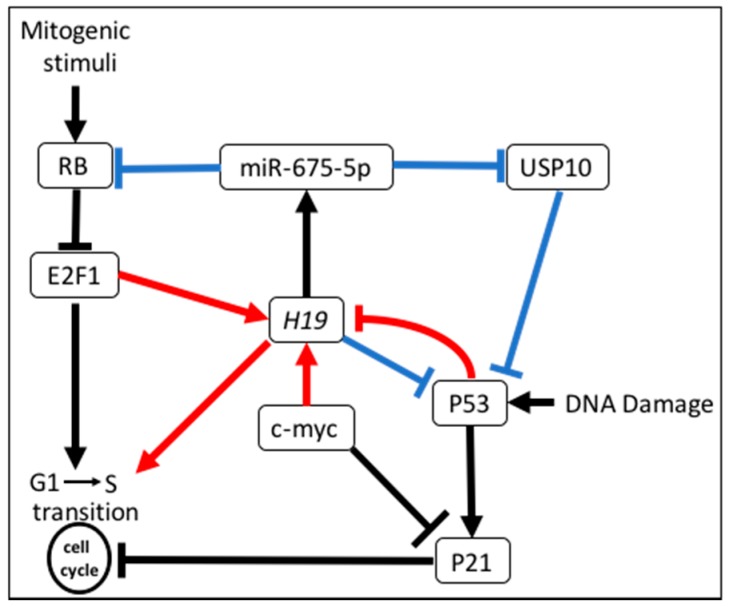
*H19* and cell cycle regulation. *H19* favors the cell cycle progression through different mechanisms. Mechanisms demonstrated in breast cancer are indicated in red, in other cellular context are indicated in blue. General mechanisms of cell cycle regulation are indicated in black. Arrows indicate positive regulations whereas lines with bars correspond to inhibition.

Besides the involvement of *H19* in EMT, migration, metastasis, and carcinogenesis through the mechanisms described above, *H19* plays a key role in the regulation of the cell cycle. The overexpression of *H19* in breast cancer cells lines facilitates cell cycle transition G1/S while downregulation of *H19* by RNA interference impedes S-phase entry and proliferation [80]. *H19* is activated by E2F1 binding (a key factor in the G1/S transition) to two consensus sites on *H19* promoter and negatively regulated by E2F6 and RB protein. Interestingly, in human colorectal cancer cell lines, *H19* through microRNA downregulates RB protein and increases cell growth [32]. Barsyte-Lovejoy et al. demonstrated that the oncogene c-Myc binds specifically to *H19* maternal allele to promote its transcription, leading to proliferation of breast and lung cancerous cells [81]. We have also shown that the tumor suppressor protein and cell cycle regulator p53 negatively regulates *H19* in tumor cells [82]. The interaction between *H19* and p53 was also described in other cancers. In gastric cancer cells *H19* physically interacts with p53 to induce p53 inactivation [83]. The *H19*-derived miR-675 negatively regulates p53 through an unknown target in bladder cancer cell [26]. In C-kit^+^ cardiac progenitor cells, Cai et al. showed that miR-675 negatively regulates p53 through the targeting of USP10 [38]. Even if the regulation of p53 by *H19* in human breast cancer is not yet described, these data, collectively, demonstrate that *H19* and miR-675 play a pivotal role in the regulation of cell cycle in cancer as illustrated in Figure 3.

## 4. Conclusions

**Figure 4:**
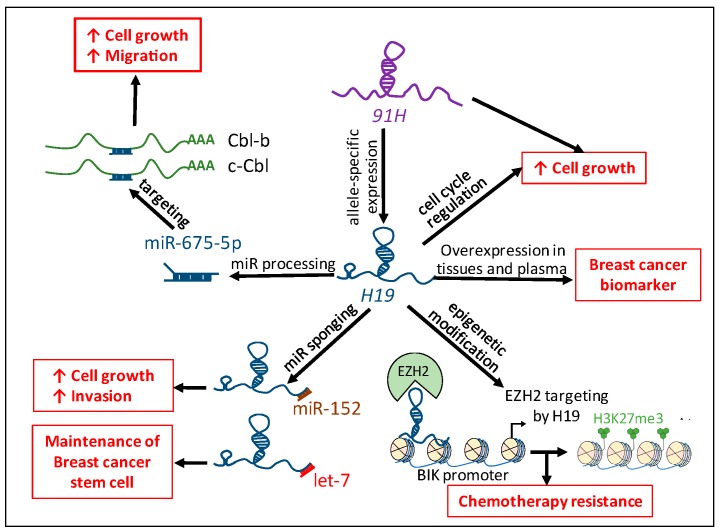
Implication of *H19* in human breast cancer. The *H19* lncRNA favors breast tumorigenicity by regulating the cell cycle, through the processing of miR-675-5p and the sponging of miR-152 and let-7, and regulating chemotherapy resistance through epigenetic modification. The *H19* antisense, *91H*, allows allele-specific expression of *H19* and participates in breast cancer cell biology. *H19* and miR-675 are overexpressed in breast cancer tissues and plasma of patients and could be used as tumor biomarkers. H3K27me3: Trimethylation of lysine 27 on histone H3. Red arrows indicate an increased phenotype.

*H19* is involved in human breast cancer through interaction with protein, microRNAs, *H19*-derived miR-675-5p, and *H19* antisense lncRNA (*91H*) but the study of these ncRNAs is not only restrained in cancer cell behavior. Increasing studies have reported their interests as cancer biomarkers and therapeutic targets. In breast cancer, Zhang et al. demonstrated that the expression of *H19* is significantly increased in cancer biopsies and plasma compared with healthy controls, plasma *H19* levels were significantly correlated with progesterone and estrogen receptors and lymph node metastasis [84]. The plasma level of *H19* is higher in patients with gastric cancer compared to normal controls. Lower *H19* expression is found in postoperative samples compared to preoperative ones [85]. Higher levels of miR-675 are also found in both tumor samples and gastric juice of patients suffering from gastric cancer [86]. The different implication of *H19* in human breast cancer is illustrated in Figure 4.

From a therapeutic perspective, a plasmid-based strategy (DTA-H19/BC-819) to target *H19* is presently in a phase 2b clinical trial for bladder cancer and in phase 1/2a for ovarian and peritoneal cancer [87,88]. Although further studies are needed, the targeting of *H19* and miR-675 could provide novel opportunities in the treatment of cancer patients.

## Figures and Tables

**Table 1:** Validated targets of miR-675-5p and miR-675-3p.

**Targets of miR-675-5p**	**Cellular Context**	**Proteins Function**	**References**
c-Cbl & Cbl-b	Breast cancer	Ubiquitin ligase E3	[33]
HDAC 4/5/6	Bone Marrow Mesenchymal Stem Cells	Histone deacetylase	[36]
CaMKIId	Cardiomyocyte hypertrophy	Serine threonine protein kinase	[37]
USP10	c-kit+ cardiac progenitor cells	Ubiquitin-specific protease	[38]
RB	Colorectal cancer	Cell cycle regulator	[32]
DDB2	Colon cancer cells	Transcriptional repressor	[39]
VDR	Colon cancer cells	Vitamin D receptor	[40]
VDAC1	Diabetic cardiomyopathy	Required for mitochondria-mediated apoptosis	[41]
REPS2	Esophageal squamous cell carcinoma	Repressor of cell proliferation and migration	[42]
CALN1	Gastric cancer	Calcium-binding protein	[43]
RUNX1	Gastric cancer	Transcription factor	[44,45]
FADD	Gastric cancer	Apoptotic adaptor that recruits caspase 8 or 10	[46]
Cadherin 13	Glioma cell	Atypical cadherin lacking the cytoplasmic domain	[47]
RB & TWIST1	Hepatocellular carcinomas	Twist1: Transcription factor	[48]
GPR55	Non-small cell lung cancer	G protein-coupled receptor	[49]
TGF-ß1	Osteoblast differenciation	Growth factor	[50]
TGF-ß1	Prostate cancer	Growth factor	[25]
NOMO1	Placental trophoblast cell	Nodal signaling pathway	[51]
ATP8A2	Skeletal cell	Catalytic component of a P4-ATPase flippase complex	[52]
CDC6	Skeletal muscle	Essential for the initiation of DNA replication	[53]
VDR	Ulcerative Colitis	Vitamin D receptor	[54]
**Targets of miR-675-3p**	**Cellular Context**	**Proteins Function**	**References**
Cadherin 11	Melanogenesis	Type II classical cadherin	[55]
MITF	Melanogenesis	Transcription factor	[56]
IGF1R	Placenta	Insulin-like growth factor 1 receptor	[57]
TGF-ß1	Osteoblast differenciation	Growth factor	[50]
SMAD1 & SMAD5	Skeletal muscle	Intracellular signal transducer and transcriptional modulator	[53]

**Table 2:** Validated miRNAs sponged by *H19*.

miRNAs Sponged by *H19*	Cellular Context	References
hsa-miR-19a/b	Acute myelocytic leukemia	[63]
miR-29b-3p	Bladder cancer	[64]
miR-152	Breast cancer	[61]
let-7	Breast cancer stem cells	[60]
miR-455	Cardiac fibrosis	[65]
let7	Endometriosis	[66]
miR-181-d	Gliobastoma	[67]
let-7	HEK293	[68]
miR-106-a & miR-17-5p	Hela Cells, myboblast	[69]
let-7	Muscle cells	[70]
let-7b & miR-200b/c	Mouse breast cancer	[62]
miR 141 miR 22	Osteoblast	[71]
mir-200s	Osteosarcoma	[72]
miR-17-5p	Thyroid cancer	[73]

## Abbreviations

RNA	Ribonucleic Acid
DNA	Deoxyribonucleic Acid
ENCODE	Encyclopedia of DNA
ncRNAs	Non-coding RNAs
lncRNas	Long non-coding RNAs
miRs	MicroRNAs
siRNAs	Small non-coding RNAs
piRNAs	PIWI-interacting RNAs
snoRNAs	Small nucleolar RNA
Her-2	Human epidermal growth factor receptor 2
PR	Progesterone receptor
ER	Estrogen receptor
IGF2	Insulin growth factor 2
BC	Breast cancer
CRC	Colorectal cancer
RB	Retinoblastoma
DNMT1	DNA Methyltransferase 1
EMT	Epithelial-mesenchymal transition
MET	Mesenchymal-epithelial transition
BIK	BCL2 Interacting Killer
NOXA	NADPH Oxidase Activator 1
MBD1	Methyl-CpG Binding Domain Protein 1
PTX	Paclitaxel
PEG1	Paternally-Expressed Gene 1 Protein
SAHH	*S*-adenosylhomocysteine hydrolase
E2F1	E2F Transcription Factor 1
CPCs	c-kit+ cardiac progenitor cells
USP10	Ubiquitin Specific Peptidase 10
